# Comparison of the effects of alcoholic extract of aerial parts of *Anvillea garcinii* and atorvastatin on the lipid profile and thyroid hormones in hypercholesterolemic rats

**DOI:** 10.22038/AJP.2021.18130

**Published:** 2022

**Authors:** Fatemeh Rasekh, Zohre Atashi-Nodoshan, Ali Zarei, Amir Abbas Minaeifar, Saeed Changizi-Ashtiyani, Zahra Afrasyabi

**Affiliations:** 1 *Department of Biology, Payame Noor University, Tehran, Iran*; 2 *Department of Physiology, Estahban School of Paramedical Sciences, School of Nursing Hazrat Zahra (P.B.U.H) Abadeh, Shiraz University of Medical Sciences, Shiraz, Iran*; 3 *Department of Physiology*,* Arak University of Medical Sciences, Arak, Iran*; 4 *Department of Midwifery, Islamic Azad university of Estahban, Shiraz, Iran*

**Keywords:** Anvillea garcinia, Atorvastatin, Cholesterol, Thyroxine, Rat

## Abstract

**Objective:**

Decreased thyroid hormones along with increased blood fats and overweight, lead to atherosclerosis and eventually cardiovascular disease. The aim of this study was to compare the effects of alcoholic extract of aerial parts of *Anvilea garcinii* and atorvastatin on the lipid profile and thyroid hormones in hypercholesterolemic rats.

**Materials and Methods:**

In this study, 40 male Wistar rats were divided into 5 groups (n=8): control, hypercholesterolemic vehicle and three experimental groups. The control group, received water and normal food daily, the hypercholesterolemic vehicle group received drug solvent (atorvastatin dissolved in distilled water) and three experimental groups received alcoholic extract of *A. garcinii* (100 and 300 mg/kg) and atorvastatin (10 mg/kg) as gavage. At the end of the 45- day period, blood samples were prepared from all groups and the amount of desired factors was measured and analyzed.

**Results:**

The amount of lipid profiles (cholesterol, HDL, LDL, VLDL,TG) and thyroid stimulating hormone in the vehicle group were increased compared to the control group, while the amount of these factors were decreased in the experimental groups compared to the vehicle group. Furthermore, the level of thyroid hormones in the hypercholesterolemic vehicle group was decreased compared to the control group, while the level of these hormones was increased in the experimental groups receiving the extract compared to the vehicle group.

**Conclusion:**

Alcoholic extract of *A. garcinii* aerial parts, may increase thyroid hormones, and sequentially reduce blood lipids; thus, it could be a good candidate for the treatment of hypercholesterolemia and hypothyroidism.

## Introduction

In the United States, more than 100 million people, or about 53% of adults, suffer from high LDL-C (low-density lipoprotein-cholesterol) and approximately 31 million people have cholesterol levels above 240 mg/L, putting them at risk for cardiovascular disease. One of the main risk factors for cardiovascular disease is hypothyroidism (Karr, 2017 ▶).

As one of the body's endocrine glands, the thyroid gland is responsible for maintaining energy homeostasis, metabolism, and stimulating cell activity, and is involved in development, differentiation, and maturation. Decreased levels of thyroid hormones reduce basal metabolism, increase fat, overweight and atherosclerosis, and eventually lead to cardiovascular disease. Therefore, the prevention of the decrease in thyroid hormones, reduces the amount of blood lipids and the risk of cardiovascular diseases. Obviously, various chemical drugs such as atorvastatin, levothyroxine, etc., which are used to reduce blood lipids and hypothyroidism cause side effects such as nausea, liver and digestive disorders, dizziness, etc. (Zabihi et al., 2020 ▶; Adibi and Khoshvaghti, 2019 ▶).

In disease prevention and treatment, the use of medicinal plants has a growing trend. In previous years, in some cases, natural medicines, especially medicinal herbs, were the only means of treatment (ShekarForosh et al., 2012 ▶). *Anvillea garcinii* is a shrub with yellow flowers that belongs to the Asteraceae family and is used in traditional medicine to treat digestive problems, hepatitis and pulmonary complications (Hammiche and Maiza, 2006 ▶; Miara, 2019 ▶). *A. garcinii *aerial parts contain phenolic compounds (Talebi et al., 2015 ▶ ), flavonoids (Perveen, 2018 ▶) and germacranolides (Abdel-Sattar and McPhail, 2000 ▶) such as 9-hydroxy parthenolide, which is widely used as a renewable source in pharmaceutical and cosmetic industries (Moumou, 2011a ▶, 2011b ▶, 2011c ▶) and several chemicals have been synthesized from it (Moumou, 2010 ▶, 2012, 2011d). As a result of its antioxidant properties, and due to its effects in terms of increasing insulin sensitivity and inhibition of α-amylase and α-glucosidase, *A. garcinii* extract has anti hyperglycemic, anti-hyperlipidemic and anti-inflammatory activities and is effective in treating diabetes and its complications (Kandouli, 2017 ▶; Kharjul et al., 2014 ▶). Consumption of *A. garcinii* ethanolic extract before CCl_4_ consumption, provides liver protection in rats (Perveen, 2018 ▶; Aqababa et al., 2016 ▶) and has a very strong anti-ulcer property (de Lira Mota, 2009 ▶; Perveen, 2018 ▶). Ethyl acetate extract of *A. garcinii* has antioxidant properties, and anticholinesterase, anti-tyrosinase and anti- α-glucosidase activity. This plant extract reduces the amount of blood lipids in diabetic rats (Abdel-Sattar and McPhail, 2000 ▶). Blood lipid levels are inversely related to thyroid hormone levels (Chin et al., 2014 ▶). Since there are few studies on hypolipidemic properties of *A. garcinii* and no reports on its effect on the pituitary gland, this study compared the effects of alcoholic extracts of aerial parts of *A. garcinii* and atorvastatin on lipid profiles and thyroid hormones in hypercholesterolemic rats.

## Materials and Methods

The present study was performed on 40 male Wistar rats with the ethical code: IR.PNU.REC.1398.117. The animals were purchased from Shiraz Vaccine and Serum Research Institute. In all stages, the approvals related to the principles of working with laboratory animals and the law on care and use of laboratory animals were observed. During the experimental period, the animals were exposed to 22 to 26°C, 12 hours of darkness and 12 hours of light and were randomly divided into 5 groups of 8 rats (Zabihi et al., 2020 ▶) as follows: 1) The control group was only on a normal diet and water; 2) The hypercholesterolemic vehicle group received daily 0.2 ml of normal saline (atorvastatin dissolved in distilled water and plant extract); 3) The hypercholesterolemic group treated with hydroalcoholic extract of the aerial parts of *A. garcinii* (0.2 ml of 100 mg / kg extract daily per rat); 4) The hypercholesterolemic group treated with hydro alcoholic extract of the aerial parts of *A. garcinii* (0.2 ml of 300 mg / kg extract daily per rat) and 5) The hypercholesterolemia group treated with atorvastatin (0.2 ml of 10 mg / kg per rat per day) (Kandouli, 2017 ▶; Kharjul et al., 2014 ▶; Perveen 2018 a ▶). The extract, normal saline and atorvastatin were administered orally. Groups 2 to 5 received a high-fat diet. All groups were starved the night before blood sampling but they had free access to water. At the end of the 45-day period, cardiac blood samples were taken for laboratory tests. The blood serum was prepared by centrifugation at 3000 rpm for 10 min and transferred to the laboratory. Then, the amount of biochemical factors such as triglyceride (TG), cholesterol, TSH (thyroid stimulating hormone), T3, T4, VLDL (very low-density lipoprotein), LDL (low-density lipoprotein) and HDL (high-density lipoprotein) were measured by a USA-made biochemical autoanalyzer (model Ra-2000) and Kit Pars tests (Pars Azmoun Co. Manufacturer of laboratory biochemistry kits under license from Diaknostic Company in Iran).

**Preparing a high-cholesterol diet**

Based on a normal rodent diet element, the high-fat diet included the rodent-based diet supplemented with 15% animal fat, 4% cholesterol (Sigma-USA) and 1% colic acid (Sigma-USA). This formula is suitable in terms of calories and energy needed to induce fatty liver. Rats were not restricted in receiving water or nutrients during the experimental period (Efati et al., 2016 ▶).

**Preparation of** ***A. garcinii*** **extract**

*A. garcinii* was collected from one of the natural habitats of this plant in Mohr city, Fars province, in April 2017 and was identified in the PNU herbarium and is kept in thePNU plant herbarium of With Herbarium code: 396026.

**Extraction by percolation method**

 To prepare the extract, after preparing the aerial parts of the plant and separating its impurities, 200 g of the plant was crushed in a mill. It was then poured slowly into the percolator. The device was filled with the powder by applying uniform pressure on the surface of the percolator. Then, a filter paper was placed on the round surface and one or two pieces of glass with a heavy metal body were placed on it, so that the uniformity of the powder would not be disturbed when adding 70% alcoholic solvent because the solvent should be added in such a way that it always covers the plant powder and does not dry out during the extraction operation. The powder was stored with the solvent for 72 hr in a closed container at room temperature. Henceforth, the valve of the device was opened and the desired extract was separated and concentrated in a rotary device at 37°C. Then, it was dried in a desiccator. Using this method, the powdered extract was obtained with a yield of 3% (Handa et al., 2008 ▶).

**Statistical analysis**

Data are reported as the mean and standard error (Mean±SD). The statistical significances were evaluated by one-way ANOVA followed by Tukey tests. These were used to do inter-group comparison, while considering p≤0.05 as the significance level.

## Results

The level of cholesterol in the hypercholesterolemia vehicle group compared with the control group, showed a significant increase. However, its level in all experimental groups compared to the hypercholesterolemia vehicle group and in experimental groups receiving *A. garcinii *extract compared to the groups receiving atorvastatin showed a significant decrease (p=0.000). The amount of HDL in the hypercholesterolemia vehicle group did not show significant changes compared to the control group. But the amount of HDL showed a significant decrease only in the experimental groups receiving high-dose (300 mg / kg) *A. garcinii* alcoholic extract compared to the hypercholesterolemia vehicle group (p=0.000). Level of TG in the hypercholesterolemia vehicle group showed a significant increase compared to the control group and its level in all experimental groups which received alcoholic extract of *A. garcinii* (100 and 300 mg/kg) and atorvastatin (10 mg/kg) compared to the hypercholesterolemia vehicle group showed a significant decrease (p=0.000). The amount of TG in the experimental group that received high-dose extract (300 mg/kg) showed a significant decrease compared to the low-dose extract group (100 mg/kg) (p=0.000). The amount of VLDL in the hypercholesterolemia vehicle group showed a significant increase compared to the control group and its level in all experimental groups which received alcoholic extract of *A. garcinii* and atorvastatin compared to the hypercholesterolemia vehicle group, showed a significant decrease. 

The level of VLDL changes in the high-dose extract group (300 mg/kg) compared to the low-dose extract group (100 mg/kg) showed a significant decrease, but its level in extract-treated groups compared to the group that received atorvastatin, did not show significant changes (p=0.000). The level of low-density lipoprotein (LDL) in the hypercholesterolemic vehicle group did not show significant changes compared to the control group but its level in the high-dose extract group (300 mg/kg) and atorvastatin group showed a significant decrease compared to the hypercholesterolemia vehicle group. Other changes in different groups of this study were not significant. Thyroxine (T4) level in the hypercholesterolemia vehicle group showed a significant decrease compared to the control group, but its level in the extract-treated groups showed a significant increase compared to the hypercholesterolemia vehicle group. The changes of this factor in the atorvastatin group were not significant compared to the hypercholesterolemia vehicle group. The amount of T4 changes in the extract-treated groups (groups received alcoholic extract of 100 and 300 mg/kg *A. garcinii*) were not significant, but its level in these groups compared to the atorvastatin group, showed a significant increase (p=0.000). The level of triiodothyronine (T3) in the hypercholesterolemia vehicle group compared to the control group did not show significant changes but its level in the extract-treated groups shows a significant increase compared to the hypercholesterolemia vehicle group. The changes of this factor in the experimental group that received atorvastatin compared to the hypercholesterolemia vehicle group, as well as in the experimental groups that received plant extracts in comparison with each other, were not significant, but the level of T3 in the experimental groups receiving *A. garcinii* extract compared to the atorvastatin group showed a significant increase (p=0.000). The level of thyroid stimulating hormone (TSH) in the hypercholesterolemia vehicle group showed a significant increase compared to the control group but its level in experimental groups that received *Anvillea garcinii* extract showed a significant decrease compared to the hypercholesterolemia vehicle group (p=0.000). The changes in this factor in the experimental group that received atorvastatin compared to the hypercholesterolemia vehicle group, as well as in the experimental groups that received the plant extract in comparison with each other, were not significant. The level of TSH in the experimental groups that received *A. garcinii* extract showed a significant increase compared to the group receiving atorvastatin (Table 1) (p=0.000).

**Table 1:** Comparison of the effects of alcoholic extract of aerial parts of *Anville agarcinii* and atorvastatin on lipid profile and thyroid hormones in hypercholesterolemic rats

Atorvastatin (10 mg/kg)	Anvilia gracini (300 mg/kg)	Anvilia gracini (100 mg/kg)	Vehicle	Control	Factors
69.00±4.13α	47.28±1.79α β	52.00±2.32α β	76.57±1.81ω	63.14 ±4.37	Cholesterol (mg/dl)
70.85±3.23α	61.42±1.86απ	78.00±6.78α	137.57±6.95ω	113.71±5.69	TG (mg/dl)
19.28±0.74α	15.71±0.42α	20.85±1.51	25.42±1.73	22.71±1.74	LDL (mg/dl)
13.14±0.59	11.00±0.43α	13.75±0.64	15.28±0.96	14.85±0.70	HDL (mg/dl)
13.0.52α	11.85±0.63απ	17.28±1.30α	27.42±1.13ω	22.00±1.25	VLDL (mg/dl)
4.90±0.68	1.71±0.19α β	2.58±0.53α β	5.90±0.27ω	3.18±0.50	TSH (ml/l)
1.95±0.0.26	3.67±029α β	3.02±0.33α	1.51±0.09	2.45±0.27	T3 (µg /dl)
5.77±0.48	9.30±0.42α β	7.97±0.45α β	4.55±0.21ω	6.0.46	T4 (µg /dl)

Each of the values represents the standard error±mean. ω: p<0.001 compared to control group; α: Significant changes in experimental groups which received alcoholic extract of *A. garcinii* and atorvastatin compared to the vehicle group (p=0.000); β: Significant changes in the experimental groups that received the plant extract compared to the atorvastatin group (p=0.000) and π: Significant differences between the plant extract treated groups (p=0.000).

## Discussion

In general, the findings of the present study show that the lipid profile including cholesterol, triglycerides and VLDL in the hypercholesterolemic vehicle group increased compared to the control group, while the lipid profile in the experimental groups that received the *A. garcinii* alcoholic extract and atorvastatin decreased. The level of thyroxine in the vehicle group decreased compared to the control group, while the level of thyroid hormones increased in the experimental groups that received the *A. garcinii* alcoholic extract. The level of TSH hormone also increased in the vehicle group, while its level decreased in the experimental groups that received the alcoholic extract of the plant. Changes in thyroid hormones and TSH in the experimental group that received atorvastatin did not show significant changes compared to the control group. 

According to the results of this study, the extract more effectively than atorvastatin, increased thyroid hormones and this effect is proportional to the dose of the extract. Also, the extract reduced TSH levels. Increased thyroid hormones can reduce TSH through a negative feedback mechanism (Zarei et al., 2014 ▶; Changizi-Ashtiyani et al., 2015 ▶).

As observed in the present study, *A. garcinii* increased thyroid hormones and decreased TSH. But in the control group where the level of thyroid hormones decreased, the level of TSH increased because it was released from the negative self-regulation mechanism of thyroid hormones. In the case of atorvastatin, although the lipid profile decreased, the amount of increase in thyroid hormones was not significant compared to other groups in this experiment. Therefore, it seems that this drug can reduce blood lipids through a mechanism of action which is different from that seen for thyroid hormones. Studies also show that atorvastatin, like other statins, is a competitive inhibitor of the enzyme HMG-COA reductase (3-hydroxyl-3-methylglutaryl-coenzyme A) and inhibits cholesterol production (Choubey et al., 2020 ▶; Talaei et al., 2018 ▶). The results of previous study also indicate the hypolipidemic effects of atorvastatin (Daniel et al., 2017 ▶). 

Thyroid hormones change the amount and activity of lipolytic enzymes, lipoprotein lipase and hepatic lipase through their effect on gene expression (Diekman et al., 1995 ▶). *Anvillea garcinii* extract also increased thyroid hormones and decreased blood lipids. Therefore, one of the mechanisms of the effects of this extract on the lipid profile is inducing an increase in thyroid hormones. The aerial parts of this plant contain two hypoglycemic compounds called 9 α-hydroxy parthenolide and 9 β-hydroxyparthenolide (Abdel-Sattar et al., 1996 ▶; Ulubelen et al., 1979). The researchers examined the antidiabetic, antioxidant and anti-inflammatory properties of *A. radiata* in high-fat diet treated rats and found that the extracts of the aerial parts of the plant had a strong dose-dependent anti-inflammatory, anti-obesity and anti-diabetic activity. Anvillea extract probably inhibits α-amylase and α-glucosidase, and due to its antioxidant properties, it scavenges free radicals and protects pancreatic beta cells (Kandouli, 2017 ▶; Kharjul et al., 2014 ▶).

Therefore, the results of the present study on the reduction of blood lipids in rats treated with Anvillea extract are not far from expectations and are in line with the results of previous studies. 

Anvillea aerial parts contain phenolic compounds (Talebi et al., 2015 ▶) and flavonoids (Perveen, 2018 a ▶). Previous research has shown that medicinal plants’ polysaccharides, flavonoids, oligo proteins, polypeptides, steroids, alkaloids and pectinin can well reduce the blood sugar and lipids (Zarei et al., 2012 ▶). Due to its antioxidant properties, increasing insulin sensitivity and inhibition of α-amylase and α-glucosidase activity, aerial parts extract of Anvillea has anti-hyperglycemic, anti-hyperlipidemic and anti-inflammatory activities and is effective in treating diabetes (Kandouli, 2017 ▶; Kharjul et al., 2014 ▶).

Blood lipid levels are inversely related to thyroid hormone levels, and as hormone levels increase, lipid levels decrease (Chin et al., 2014 ▶). Anvillea extract also increased thyroid hormones and decreased blood lipids. As a result, one of the possible mechanisms of the effects of this extract on the lipid profile is inducing an increase in thyroid hormones. With the use of atorvastatin, although the lipid profile decreased, but the amount of thyroid hormones increase was not significant. Atorvastatin inhibits the enzyme HMG-COA reductase and ultimately inhibits cholesterol production (Choubey et al., 2020 ▶; Talaei et al., 2018 ▶). Hydroalcoholic extract of *A. garcinii* increased thyroid hormones more effectively than atorvastatin. Alcoholic extract of this plant also reduced TSH and lipid profile in hypercholesterolemic rats. Due to the relationship between thyroid hormones and blood lipid levels with some neurotransmitters, it is recommended to study the effects of *A. garcinii* extract on dopamine and leptin levels and obesity gene expression.

## Conflicts of interest

The authors have declared that there is no conflict of interest.
